# Three-Dimensional Finite Element Analysis of En Masse Retraction With Integration of Maxillary Anterior Teeth

**DOI:** 10.7759/cureus.85388

**Published:** 2025-06-05

**Authors:** Yoshikazu Ishii, Hiroya Ozaki, Kenji Fushima

**Affiliations:** 1 Division of Orthodontics, Department of Highly Advanced Stomatology, Graduate School of Dentistry, Kanagawa Dental University, Yokosuka, JPN

**Keywords:** anchor screw, edgewise multi-bracket appliance, en masse retraction, finite element analysis, maxillary anterior teeth

## Abstract

Background: As a novel force system to safely and efficiently retract the maxillary anterior teeth in orthodontic treatment, we applied an en masse traction method with integration of the maxillary anterior teeth (EMTI) and quantitatively evaluated tooth movement using a three-dimensional (3D) finite element analysis to examine the validity of EMTI.

Methods: A 3D finite element model (FEM) of the six teeth of the maxillary dentition, periodontal ligament, and alveolar bone was created. A two-tooth model of the bilateral central incisors, a four-tooth model of the bilateral central and lateral incisors, and a six-tooth model of the bilateral central and lateral incisors and canines were reconstructed as EMTI models. Each tooth was splinted to the other with a palatally attached wire, and the moment arms were attached to this wire and extended apically. Traction points were set along the moment arms at 6, 8, and 10 mm from the level of the palatal wire. FEM analysis was performed by applying a traction force of 1.0 N in the palatal direction to the traction point of each moment arm. For the maxillary central incisors of each model, the angular changes in the tooth axis and center of rotation (C-Rot) were investigated. In addition, the 3D displacements of the crown and apex of each tooth were analyzed for each EMTI model.

Results: In the two-tooth and four-tooth models, traction of 6 mm showed palatal inclination of the tooth axis, and the C-Rot was located near the root apex. Traction by 8 mm showed bodily tooth movement, and traction of 10 mm demonstrated labial inclination of the tooth axis, in which the crown was displaced labially and the root was displaced palatally. In the six-tooth model, traction by 10 mm exhibited en masse palatal movement of all six teeth without inducing a bowing effect at the canines.

Conclusions: FEM analysis of the EMTI technique provided useful information for understanding the 3D movement of the root in clinical practice.

## Introduction

In cases of maxillary protrusion, the first premolars and other teeth are extracted to eliminate discrepancies between the jaw size and crown width. The extracted space is generally closed using an edgewise multi-bracket appliance (MBA) for palatal movement of the upper anterior teeth. There are two main methods for loops and sliding mechanics. The conventional method involves distal movement of the canines followed by palatal movement of the central and lateral incisors. The loops are incorporated into the arch wire at the sites of space closure, and when using sliding mechanics, brackets are placed along the arch wire. However, sliding mechanics create friction between the brackets and arch wires, preventing teeth from moving efficiently [1].

It is difficult to control the three-dimensional (3D) movement of the root sufficiently using a force system applied by a commonly used MBA [2]. Orthodontic force is applied to the bracket attached to the crown, which is distant from the center of resistance (C-Res). The C-Res for tooth movement is thought to be located near the crown at one-third of the root length [1]. Therefore, when moving the upper anterior teeth palatally, the force applied to the bracket causes palatal inclination of the tooth axis, which is clinically referred to as uncontrolled tipping [3-6]. Uncontrolled tipping causing excessive palatal inclination of the maxillary anterior teeth may impair the esthetics of the anterior teeth [2]. Uncontrolled tipping is often accompanied by labial movement of the root apex and may consequently cause complications, such as apical root resorption and alveolar bone dehiscence [7]. Reports are available on the addition of long arms to the labial and palatal main arches of an MBA, effects of changes in the traction direction, and use of orthodontic anchor screws as a fixation source [3-6,8-12].

As a novel force system to safely and efficiently retract the maxillary anterior teeth, we applied an en masse traction method with integration of the maxillary anterior teeth (EMTI) instead of an MBA. Multiple maxillary anterior teeth were integrated with a stainless steel wire attached on the palatal aspect, and an orthodontic force was applied to the tip of the moment arm, extending in the apical direction to pull the teeth into a group. An orthodontic anchor screw implanted deep in the palate is used as a source of traction anchorage [13], and it has become possible to control the traction force and direction more freely than ever before [8,9,11,12].

Few reports exist on the methods of directly connecting multiple teeth and pulling them together without using brackets; nevertheless, Jang et al. and Kim et al. have reported on the clinical effectiveness of these methods [14,15].

Controlled tipping through EMTI may involve safe tooth movement. In addition, the friction between the bracket and wire when using an MBA is not present during EMTI. For efficient and safe controlled tipping of the maxillary anterior teeth using single-lump traction, it is necessary to simulate the 3D movement of the tooth root. In this study, we quantitatively evaluated tooth movement using a 3D finite element analysis to examine the validity of EMTI.

This article was previously posted to the Research Square preprint server on February 7, 2024.

## Materials and methods

A 3D finite element model (FEM) of the maxillary dentition consisting of six teeth (bilateral central incisors, lateral incisors, and canines) was created using Fusion360 (Autodesk, Inc.), with reference to the anatomical morphology, such as the average crown width and root length [16]. The periodontal ligament (PDL) with 0.3 mm thickness and the alveolar bone were also modeled [6].

**Figure 1:**
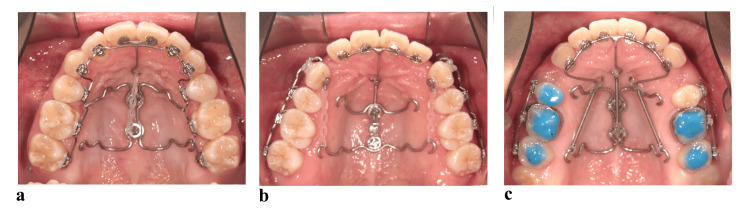
Multiple maxillary anterior teeth were integrated with a stainless steel wire attached to the palatal aspect, and an orthodontic force was applied to the tip of the moment arm, extending in the apical direction to pull them into a group. (a) Two-tooth traction in clinical practice. (b) Four-tooth traction in clinical practice. (c) Six-tooth traction in clinical practice.

**Figure 2:**
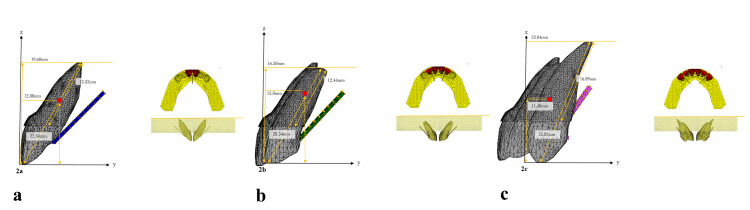
The 3D finite element model. (a) Two-tooth model consists of 114,847 isotropic tetrahedral solid elements and 28,285 nodes. Incisal edge–apex (vertical): 19.60 mm, incisal edge–apex: 22.54 mm, incisal edge–center of resistance: 12.08 mm, apex–periodontal ligament: 13.52 mm, and moment arm: 6, 8, 10 mm. (b) Four-tooth model consists of 148,263 isotropic tetrahedral solid elements and 37,702 nodes. Incisal edge–apex (vertical): 16.50 mm, incisal edge–apex: 20.34 mm, incisal edge–center of resistance: 12.00 mm, apex–periodontal ligament: 12.44 mm, and moment arm: 6, 8, 10 mm. (c) Six-tooth model consists of 184,554 isotropic tetrahedral solid elements and 48,244 nodes. Incisal edge–apex (vertical): 23.04 mm, incisal edge–apex: 25.82 mm, incisal edge–center of resistance: 11.40 mm, apex–periodontal ligament: 16.89 mm, and moment arm: 6, 8, 10 mm.

Three types of EMTI models were prepared for the FEM analysis (Figures 1, 2). A two-tooth model of the bilateral central incisors (Figure 2a), a four-tooth model of the bilateral central and lateral incisors (Figure 2b), and a six-tooth model of the bilateral central and lateral incisors and canines (Figure 2c) were constructed. Each tooth was integrated using a wire attached to the palatal surface. The moment arms were attached to the wire and extended apically. The palatal wire and moment arm were made of 0.9 mm diameter stainless steel wires. Each tooth model was an isotropic tetrahedral element. The two-tooth model consisted of 114,847 elements and 28,285 nodes, the four-tooth model had 148,263 elements and 37,702 nodes, and the six-tooth model had 184,554 elements and 48,244 nodes. A 3D solid model was created using Meshmixer (Autodesk, Inc.) and Fu-sion360 (Autodesk, Inc.), and the FEM analysis was performed using Marc/mentat (MSC soft-ware Corporation) [6]. The material conditions used in this study are listed in Table 1. The model was restrained in 6 degrees of freedom at the bottom of the bone to avoid sliding movement of the entire model. Contact boundary conditions were assumed so that tooth, PDL, and bone could be in contact with each other.

**Table 1:** Material parameters of tooth, PDL, alveolar bone, and moment arm PDL: Periodontal ligament.

	Young's modulus (MPa)	Poisson's ratio
Tooth	20,000	0.30
PDL	0.05	0.30
Alveolar bone	2,000	0.30
Moment arm	200,000	0.30
Lingual wire	200,000	0.30

A coordinate system based on the occlusal plane was used to analyze the 3D displacement of the teeth. The occlusal plane was determined using three points: the crown points of the left and right first molars and the midpoint of the incisal edges of the left and right central incisors. As shown in Figure 2, the xy-plane represents the occlusal plane, the y-axis represents the midsagittal line of the occlusal plane, and the z-axis is perpendicular to the xy-plane.

**Figure 3:**
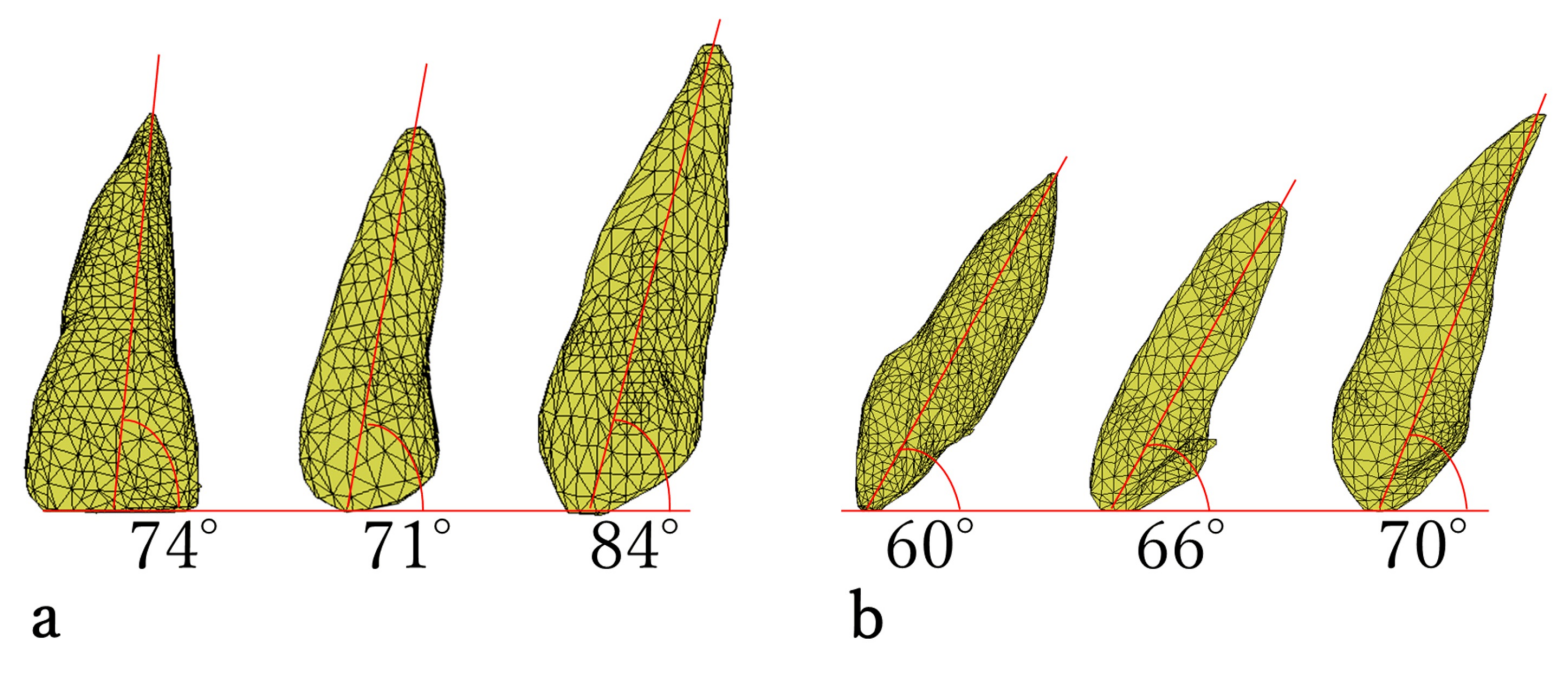
Tooth axis angle. (a) Tooth axis angle in the frontal plane view. From the left in the model, the order is central incisor, lateral incisor, and canine. (b) Tooth axis angle in the lateral plane view. From the left in the model, the order is central incisor, lateral incisor, and canine.

**Figure 4:**
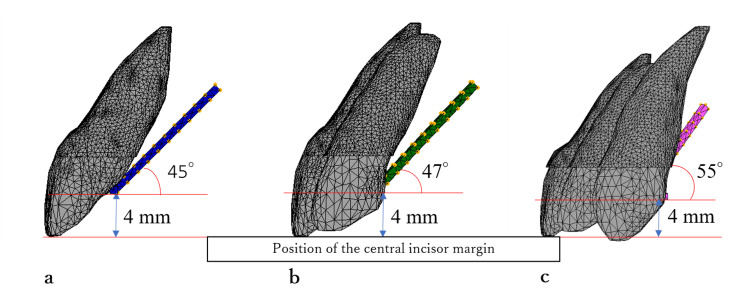
Moment arm of maxillaary anterior integrated model. (a) Two-tooth model. (b) Four-tooth model. (c) Six-tooth model.

Figure 3 shows the axial angles of each tooth in the frontal and sagittal planes [16]. Figure 4 shows the state of the moment arm attached to the palatal aspect in each model. The palatal wire was placed 4.0 mm apical to the incisal edge of the central incisor. In the two-tooth model, a moment arm was attached to the midline of the palatal wire at a 45° inclination to the occlusal plane. In the four-tooth model, two moment arms were attached to the palatal wire between the central and lateral incisors bilaterally at a 47° inclination to the occlusal plane. In the six-tooth model, two moment arms were attached to the palatal wire between the lateral incisors and canines bilaterally at an inclination of 55° to the occlusal plane.

Traction points were set along the moment arms at z-coordinates of 6, 8, and 10 mm from the palatal wire. The FEM analysis was performed by applying a traction force of 1.0 N in the palatal direction (parallel to the y-axis) to the traction point of each moment arm.

The angular change in the axis (U1-I) of the maxillary central incisor of each model was investigated. The center of rotation (C-Rot) was considered the intersection of the original tooth axis and the tooth axis following palatal traction, and the y- and z-coordinates were calculated. In addition, the 3D displacements of the crown and apex of each tooth were analyzed for each EMTI model.

## Results

**Figure 5:**
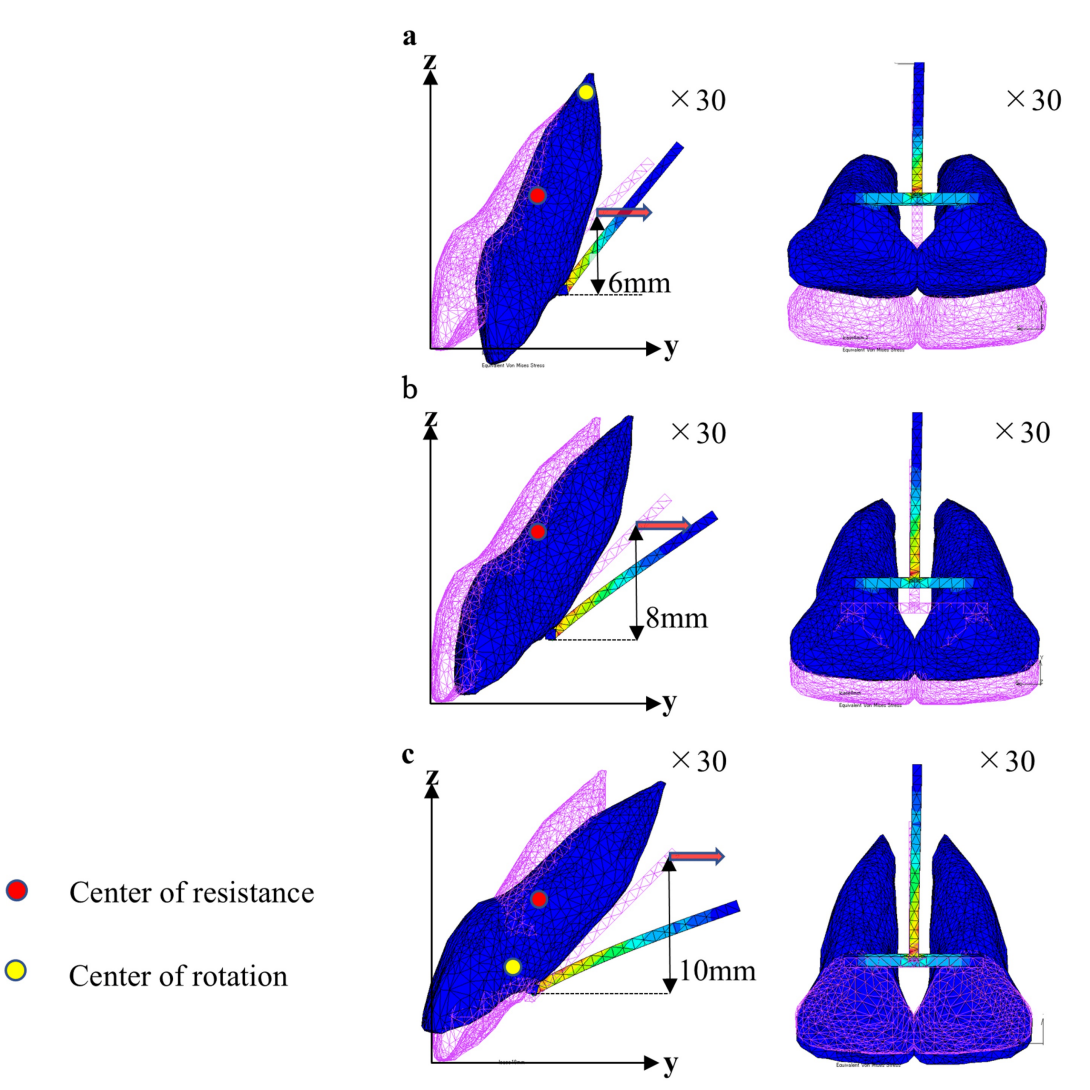
Movement of the maxillary central incisor (two anterior teeth) with moment arm from 6 mm to 8 mm (sagittal plane view, occlusal plane view). Moment arm: (a) 6 mm, (b) 8 mm, and (c) 10 mm.

**Figure 6:**
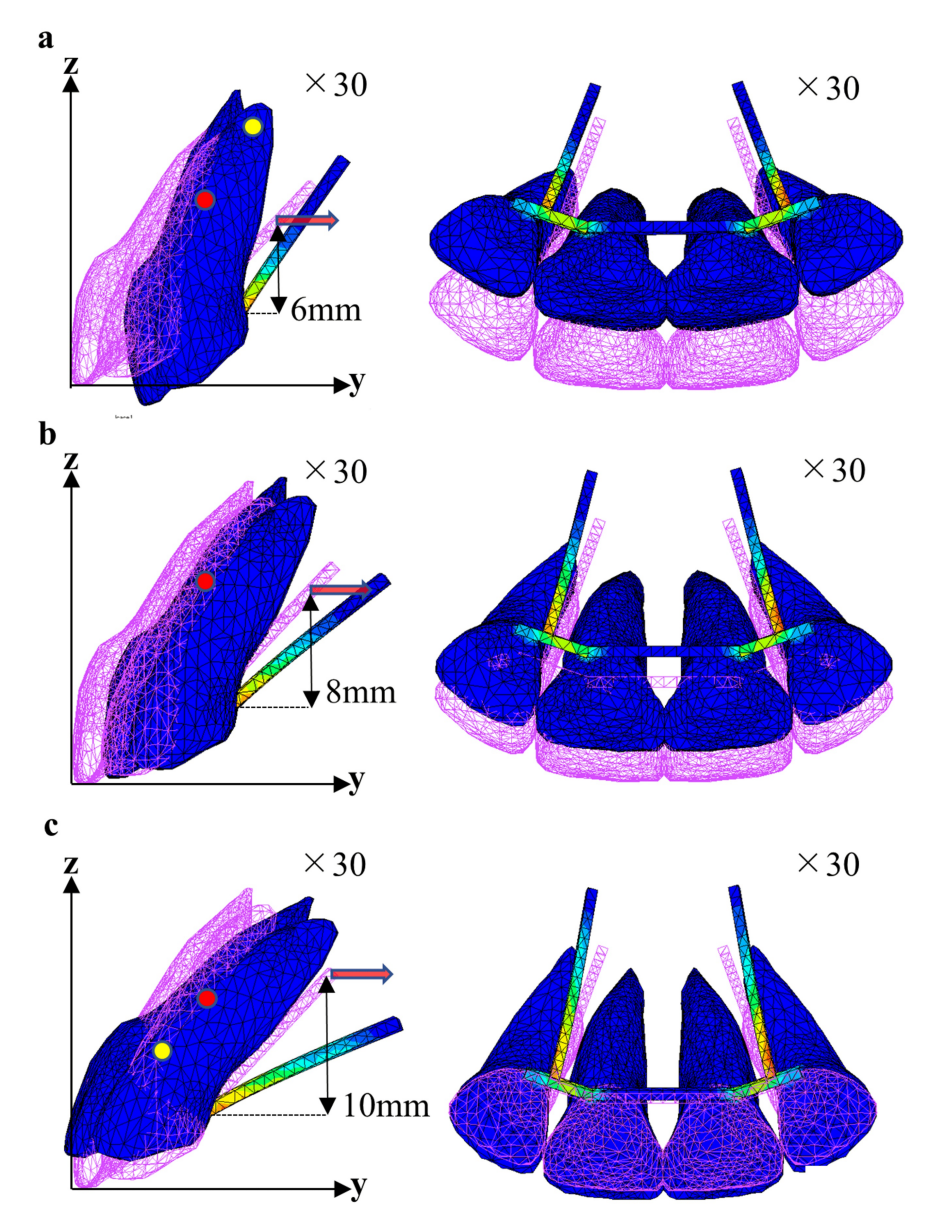
Movement of the maxillary central incisor (four anterior teeth) with moment arm from 6 mm to 8 mm (sagittal plane view, occlusal plane view). Moment arm: (a) 6 mm, (b) 8 mm, and (c) 10 mm.

**Figure 7:**
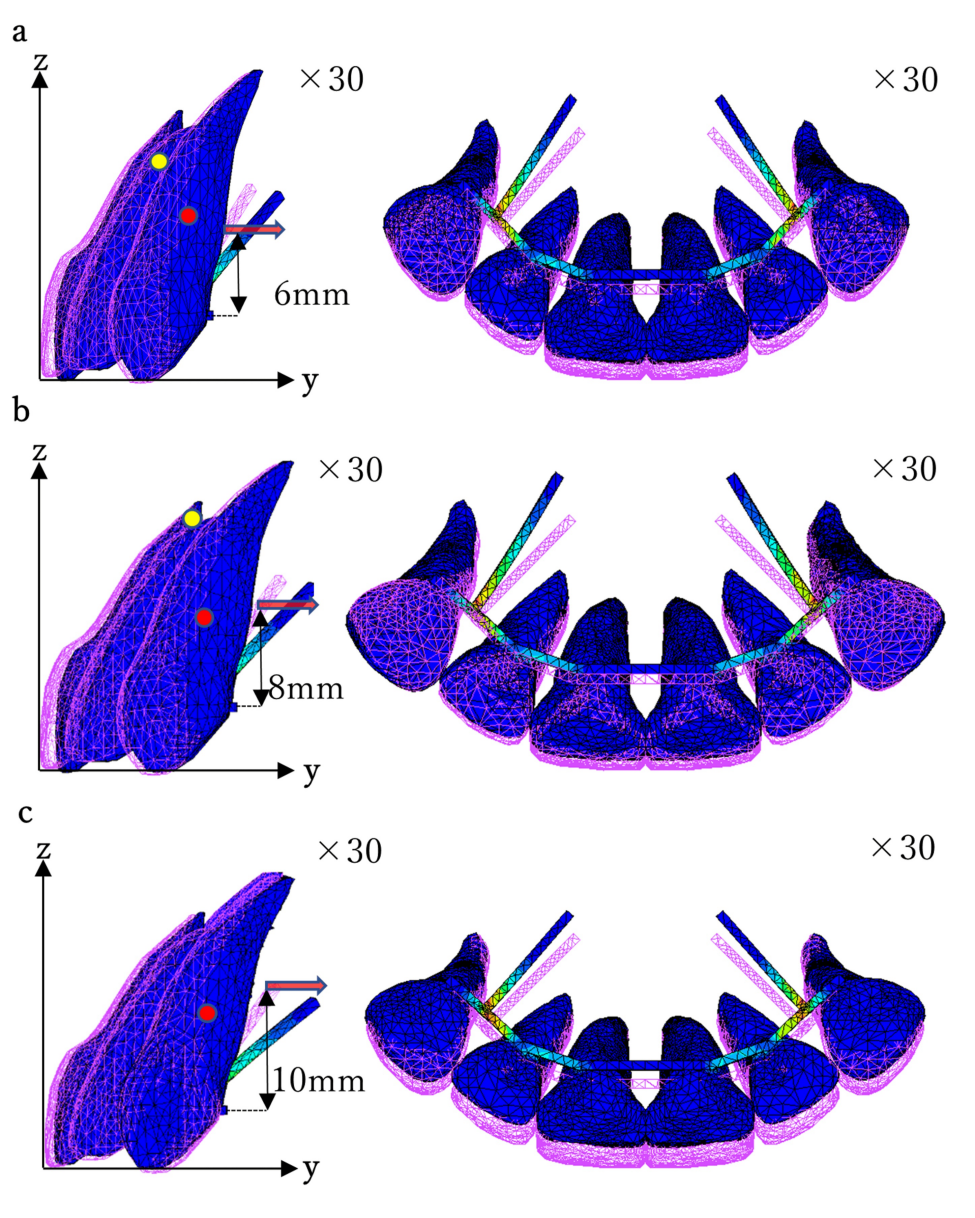
Movement of the maxillary central incisor (six anterior teeth) with moment arm from 6 mm to 8 mm (sagittal plane view, occlusal plane view). Moment arm: (a) 6 mm, (b) 8 mm, and (c) 10 mm.

Figures 5-7 show the displacement pattern in the two-tooth model, four-tooth model, and six-tooth model, respectively, with the traction level on the moment arm set to 6, 8, and 10 mm. To clearly demonstrate the traction results, the tooth displacement was evaluated 30 times.

Similar displacement patterns are seen in the two-tooth and four-tooth models. Traction by 6 mm resulted in palatal inclination of the tooth axis with palatal displacement of the crown. Traction by 8 mm showed bodily movement with lingual displacement of the crown and root. On the other hand, traction by 10 mm demonstrated a labial inclination of the tooth axis, in which the crown was displaced labially and the root was displaced palatally (Figures 5, 6).

In the six-tooth model, retraction by 6 mm and 8 mm resulted in mild palatal inclination of the tooth axis and palatal crown displacement. Traction by 10 mm showed an almost en masse palatal displacement (Figure 7).

The angular change in the axis of the central incisor (U1-I) was investigated in the sagittal plane (Table 2). In the two-tooth and four-tooth models, traction by 8 mm showed 0.02° U1-I, indicating a bodily movement palatally. When the traction level of the moment arm was shorter than 8 mm, palatal tipping occurred, and when it was longer, labial tipping occurred (Figures 5, 6). Traction by 6 mm revealed a positive U1-I of 0.18° in the two-tooth model and of 0.45° in the four-tooth model. On the other hand, traction at 10 mm revealed a negative U1-I of -0.21° in the two-tooth model and of -0.47° in the four-tooth model. In the six-tooth model, mild palatal tipping was observed at a moment-arm traction level of 6 and 8 mm with U1-I values of 0.19° and 0.09°, respectively. The traction at 10 mm led to a U1-I value of 0°, indicating bodily tooth displacement (Figure 7c).

The C-Rot was also investigated in the sagittal plane (Table 2). Figures 5-7 present the C-Rot and C-Res for each EMTI model. When palatal traction was applied to the moment arm at the 6-mm level in the two-tooth and four-tooth models, the C-Rot was located near the root apex (Figures 5a, 6a). The coordinates of the C-Rot in the two-tooth model were 10.86 mm and 18.84 mm along the y- and z-axes, respectively. In the four-tooth model, the coordinates of the C-Rot were 10.32 mm and 17.90 mm along the y- and z-axes, respectively. When 8-mm traction was applied in the two-tooth and four-tooth models, the C-Rot was far away from the C-Res (Figures 5b, 6b), indicating bodily tooth movement. When 10-mm traction was applied in the two-tooth and four-tooth models, the C-Rot was located near the level of the palatal wire (Figures 5c, 6c). The coordinates of the C-Rot were 3.43 mm and 5.96 mm along the y- and z-axes, respectively, in the two-tooth model, and 3.60 mm and 6.24 mm along the y- and z-axes, respectively, in the four-tooth model.

**Table 2:** Central incisal edge and root apex shift before and after maxillary anterior integrated traction ^a^+: Lingual direction; -: labial direction. ^b^+: Intrusion; -: extrusion.

Model	Moment arm	Shift	y-coordinate^a^	z-coordinate^b^
Two-tooth model	6 mm	Incisal edge	0.067	-0.018
		Root apex	0.006	0.017
	8 mm	Incisal edge	0.027	0.005
		Root apex	0.033	0.002
	10 mm	Incisal edge	-0.012	0.029
		Root apex	0.059	-0.013
Four-tooth model	6 mm	Incisal edge	0.159	-0.048
		Root apex	0.005	0.040
	8 mm	Incisal edge	0.066	0.012
		Root apex	0.072	0.001
	10 mm	Incisal edge	-0.027	0.071
		Root apex	0.134	-0.022
Six-tooth model	6 mm	Incisal edge	0.062	-0.024
		Root apex	-0.002	0.012
	8 mm	Incisal edge	0.083	-0.037
		Root apex	-0.022	0.025
	10 mm	Incisal edge	0.022	0.006
		Root apex	0.022	0.006

In the six-tooth model, following a traction by 6 mm, the C-Rot was located near the apex at one-third of the root length (Figure 7a), and the coordinates were 8.41 mm and 14.59 mm along the y- and z-axes, respectively. When 8-mm traction was performed, the C-Rot was located near the root apex and was accompanied by palatal tipping (Figure 7b). The coordinates of the C-Rot were 10.06 mm and 17.45 mm along the y- and z-axes, respectively. In addition, traction by 10 mm showed bodily tooth movement (Figure 7c).

Table 3 presents the displacements of the incisal edge and root apex of the central incisor for each EMTI model and traction level. Because the C-Rot was near the root apex following traction by 6 mm in the two-tooth and four-tooth models (Figures 5a, 6a), slight palatal displacement of the root apex of 0.006 mm and 0.005 mm, respectively, and intrusion of 0.017 mm and 0.04 mm, respectively, were observed. Traction by 8 mm in the two-tooth and four-tooth models (Figures 5b, 6b) revealed bodily movement. The incisal tip and root apex showed similar palatal displacements of 0.027 mm and 0.033 mm, respectively, in the two-tooth model and of 0.066 mm and 0.072 mm, respectively, in the four-tooth model. The incisal edge and root apex were displaced in opposite directions when palatal retraction by 10 mm was simulated in the two- and four-tooth models. In the two-tooth model, the incisal edge was displaced labially by -0.012 mm, and the root apex was displaced palatally by 0.059 mm. In the four-tooth model, the incisal edge was displaced labially by -0.027 mm, and the root apex was displaced palatally by 0.134 mm.

**Table 3:** Center of rotation coordinates (y-coordinate, z-coordinate), inclination angle of maxillary central incisor ^a^+: Lingual direction. ^b^+: Root apex direction. ^c^+: Lingual incline; -: labial tipping.

Model	Moment arm (mm)	C-Rot (mm)		Central incisor inclination angle (°)^c^
		y^a^	z^b^	
Two-tooth model	6	10.86	18.84	0.18
	8	-37.33	-64.75	0.02
	10	3.43	5.96	-0.21
Four-tooth model	6		17.90	0.45
	8	-74.54	-129.28	0.02
	10	3.60	6.24	-0.47
Six-tooth model	6	8.41	14.59	0.19
	8	10.06	17.45	0.09
	10	-1566.30	-2716.48	0

On simulating palatal retraction of 8 mm in the six-tooth model, the incisal edge was displaced palatally by 0.083 mm, and the root apex was displaced labially by -0.022 mm, indicating uncontrolled tipping. This movement was accompanied by extrusion of -0.037 mm. EMTI was demonstrated when palatal retraction of 10 mm was simulated in the six-tooth model. The incisal edge and root apex were displaced in the same direction, with a palatal displacement of 0.022 mm and intrusion of 0.006 mm.

Table 4 lists the canine cusp and root apex displacements in the six-tooth model. Because the canines are located at the curvature of the dental arch, their movement on the yz-plane was mesio-distal rather than bucco-palatal. The root apex was displaced distally by 0.005 mm for 6-mm traction, 0.021 mm for 8-mm traction, and 0.038 mm for 10-mm traction. Traction at 10 mm showed mesial displacement of the cusp. Regarding the vertical displacement of the root apex, intrusion of 0.021 mm was found on applying 6-mm traction and of 0.007 mm on applying 8-mm traction; on the other hand, extrusion of -0.007 mm was found with 10-mm traction.

**Table 4:** Canine cusp before and after maxillary anterior integrated traction, and root apex shit: six-tooth model (canine cusp, root apex shift） ^a^+: Lingual direction; -: labial direction. ^b^+: Intrusion; -: extrusion.

Moment arm	Shift	y-coordinate^a^	z-coordinate^b^
6 mm	Incisal edge	0.041	0.002
	Root apex	0.005	0.021
8 mm	Incisal edge	0.014	0.007
	Root apex	0.021	0.007
10 mm	Incisal edge	-0.013	0.013
	Root apex	0.038	-0.007

## Discussion

**Figure 8:**
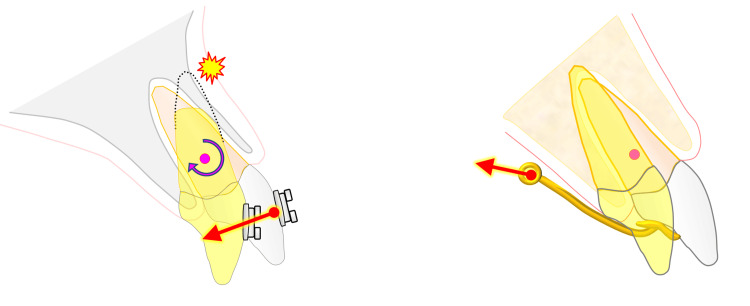
Comparison of moving the upper anterior teeth palatally. (a) Uncontrolled tipping. (b) Controlled tipping. This figure is an original work of the authors.

MBAs are commonly used for orthodontic treatment after complete eruption of the permanent teeth. During tooth movement, orthodontic force is transmitted to the periodontal tissue through a bracket attached to the crown. Orthodontic treatment involves appropriate positioning of the roots in the supporting periodontal tissue, and therefore, 3D root control is important when using an MBA (Figure 8). However, it is difficult to appropriately control the root in three dimensions because the orthodontic force is applied to the bracket attached to the crown, which is far from the C-Res [2].

Root control is particularly difficult in cases of upper protrusion in which the maxillary anterior teeth are excessively inclined labially. Palatal traction of the maxillary anterior teeth to improve protrusion tends to result in the loss of root control, leading to excessive palatal inclination of the tooth axis, known as uncontrolled tipping [3-6]. While the crowns of the incisors move palatally, the root apex may move labially, closer to the labial cortical bone. On the other hand, excessive palatal movement of the root can cause the apex to compress the palatal cortical bone. The compressive movement of the root toward the labial or palatal cortical bone may cause apical root resorption [17]. In a report on root resorption in patients who underwent orthodontic treatment with an edgewise MBA, severe root resorption exceeding one-fourth of the root length occurred in the maxillary incisors, and moderate or greater root resorption was observed in the maxillary and mandibular incisors [1]. If extensive palatal retraction of the maxillary anterior teeth is planned, careful attention should be paid to the 3D control of the roots.

In clinical practice, the bracket-wire system has limitations in retracting the maxillary anterior teeth without causing incidental symptoms, such as apical root resorption. As an alternative to an MBA, we attempted to apply EMTI using anchorage devices implanted in the palate. The friction between the bracket and wire of the MBA can be avoided using EMTI, and the orthodontic mechanics can be simplified. In addition, for controlled tipping, a long traction moment arm was attached palatally and extended apically to create a counter moment. Lingual movement progressed with a gentle orthodontic force of approximately 50 g per 1 cm surface area of the root. Therefore, if the 3D movement of roots can be controlled, efficient and safe orthodontic treatment can be administered. En masse traction of the maxillary anterior teeth, similar to EMTI, has been described before [14,15]; however, the design and mechanics of the appliances are different. To position EMTI as a clinically safe and efficient orthodontic method, it is necessary to perform further simulations in different conditions related to the number of teeth to be integrated, the design of the device, and the direction of traction. To simulate different clinical scenarios in the present study, the FEM analysis of EMTI was performed on standard models of the anterior teeth with different numbers of teeth to be integrated and heights of the moment arm.

In the two-, four-, and six-tooth models set up in this study, we investigated the effect of different traction heights of the moment arm on the inclination of the tooth axis and the displacement of the crown and root apex.

In the two-tooth and four-tooth models, traction by 6 mm showed a palatal inclination of the central incisors, suggesting controlled tipping (Figures 5a, 6a). Applying palatal retraction force to the crown side of the theoretical C-Res causes palatal inclination. As the C-Rot was located almost at the root apex, the labiopalatal displacement of the root apex was small (Table 3), and the risk of apical root resorption was considered low. Traction by 8 mm induced a force to the palatal region through the C-Res, resulting in bodily tooth movement (Figures 5b, 6b). Traction by 10 mm caused labial inclination of the central incisors. The root was largely palatally displaced, whereas the crown was labially displaced. Excessive palatal movement of the root apex may induce root resorption. Therefore, caution should be exercised.

In the six-tooth model, traction by 6 and 8 mm showed a slight palatal inclination of the central incisors, accompanied by labial displacement of the root apex. Traction of 10 mm exhibited en masse palatal movement (Figure 7, Table 3). The displacement patterns in the six-tooth model differed from those in the two-tooth and four-tooth models. This may be due to the differences in the 3D arrangement of the roots within the alveolar bone. On the sagittal view, the four-tooth model was thought to be similar to the two-tooth model. In the six-tooth model, because the canines are located at the curvature of the dental arch, the 3D arrangement of the teeth differed significantly from those in the two-tooth and four-tooth models, and the resistance form to the palatal traction was expected to be different.

The bowing effect associated with distal movement of the canines is an important issue related to space closure following extraction of the first premolar when using an MBA [18]. To perform EMTI in a six-tooth model, the canines should be moved distally toward the extraction site without inducing a bowing effect. Among the anterior teeth, the canine has the longest and largest root, making it difficult to control during orthodontic movement. The resistance of the canine to distal movement is thought to substantially affect the movement of all six anterior teeth. The key to efficient EMTI is the distal movement of the canine root. When 10 mm retraction was applied to the six-tooth model, the root apex was displaced distally, whereas the crown cusp was displaced mesially, which is opposite to the bowing effect.

In clinical practice, there are large individual differences in the root morphology, length, and tooth axis. Therefore, the number and position of the moment arms in EMTI and the height and direction of traction are subject to clinical trial and error. Finite element analysis of EMTI should be further studied to propose protocols for clinical practice.

There are major limitations in this study that could be addressed in future research. In this study, the 3D displacements of the crown and apex of each tooth were analyzed for each EMTI model. Future research should focus on the PDL stress distribution by varying the retraction of EMTI applied to the maxillary anterior teeth.

## Conclusions

In the two-tooth and four-tooth models, traction by 6 mm resulted in palatal inclination of the tooth axis, and the C-Rot was located near the root apex. Traction by 8 mm showed bodily tooth movement, and traction by 10 mm demonstrated a labial inclination of the tooth axis, in which the crown was labially displaced and the root was palatally displaced.

In the six-tooth model, traction by 10 mm exhibited en masse palatal movement of all six teeth without inducing a bowing effect on the canines. Our FEM analysis of EMTI provides useful information to understand the 3D movement of tooth roots in clinical practice.
